# High-resolution traction force microscopy on small focal adhesions - improved accuracy through optimal marker distribution and optical flow tracking

**DOI:** 10.1038/srep41633

**Published:** 2017-02-06

**Authors:** Claude N. Holenstein, Unai Silvan, Jess G. Snedeker

**Affiliations:** 1 Biomechanics Laboratory, University Hospital Balgrist, University of Zurich, 8008 Zürich, Switzerland; 2 Institute for Biomechanics, ETH Zurich, 8008 Zürich, Switzerland

## Abstract

The accurate determination of cellular forces using Traction Force Microscopy at increasingly small focal attachments to the extracellular environment presents an important yet substantial technical challenge. In these measurements, uncertainty regarding accuracy is prominent since experimental calibration frameworks at this size scale are fraught with errors – denying a gold standard against which accuracy of TFM methods can be judged. Therefore, we have developed a simulation platform for generating synthetic traction images that can be used as a benchmark to quantify the influence of critical experimental parameters and the associated errors. Using this approach, we show that TFM accuracy can be improved >35% compared to the standard approach by placing fluorescent beads as densely and closely as possible to the site of applied traction. Moreover, we use the platform to test tracking algorithms based on optical flow that measure deformation directly at the beads and show that these can dramatically outperform classical particle image velocimetry algorithms in terms of noise sensitivity and error. We then report how optimized experimental and numerical strategy can improve traction map accuracy, and further provide the best available benchmark to date for defining practical limits to TFM accuracy as a function of focal adhesion size.

Recent findings in the research field of cell biomechanics have shown that the physical forces exerted by cells to their surrounding provide a crucial feedback for cell adhesions, growth, differentiation, migration and other key cellular functions^1^,^2^,^3^,^4^. These forces are generated by the actin-myosin complex of the cytoskeleton and act on the surrounding matrix of the cell through intercellular protein complexes called focal adhesions (FA).

To estimate the magnitude and direction of these forces, indirect methods commonly referred to as Traction Force Microscopy (TFM) are used, which are based on the coupling between cell-generated traction forces and the corresponding deformation of the surrounding matrix. This approach usually involves three separate steps: 1. Imaging fluorescent beads embedded in a synthetic substrate before and after cellular tension has been released (e.g. cell detachment or drug treatment), 2. Calculating the deformation caused by the cells by tracking the beads and 3. Transformation of these displacements into traction forces using a mechanical model of the underlying substrate.

Although many variants of the original TFM method have been developed in recent years, their utility highly depends on the experimental setup, the imaging and the choice of methods and parameters for both displacement measurement and force reconstruction. As the TFM workflow requires that results of one step are fed as input to the subsequent function, error propagation results in a very high sensitivity of the system to noise with a potentially large influence on the results. For quantitative analysis of the results, it is indispensable to trace the contribution of each factor to the overall result (Fig. 1). Despite many experimental efforts, very little research has been done to adequately benchmark analytical approaches including all three sub-steps, as a representative simulation and calibration environment has yet to be fully established^5^.

On different occasions, it has been shown that even “best-practices” for TFM can be very unstable in terms of sensitivity to input parameters. The main consequence is that methods often drastically underestimate true traction forces and focal adhesion sizes. Among the primary sources of errors, this underestimation of traction mostly results from resolution loss during displacement field calculation which may reduce the peak stress up to 50%^6^,^7^, depending on the size of the focal adhesion. On the other hand, the inversion of the elastic equation that is needed to calculate the traction field is an ill-posed problem, meaning that small variations in the displacement data and experimental noise can provoke large differences in the outcome of the traction calculation^8^,^9^. This makes the classic TFM approach highly sensitive to experimental noise and tracking errors.

Many published studies have tried to mitigate such numerical instability by filtering the displacement data to remove high-frequency signal components^10^, suppressing the noise amplification in the process of traction reconstruction by using filters based on optimal signal processing theory^11^, or by constraining the solution of the equation by using a regularization scheme^6^,^12^. Despite all efforts to mitigate corruptive effects of noise in displacement data during force reconstruction, careful review of the literature reveals that little attention has until now been paid to the image acquisition strategy and accuracy of the displacement field calculation itself.

To enable a quantitative optimization of the TFM outcome, we developed a novel cell traction force simulation and evaluation platform based on finite element analysis (FEA) with which the complete TFM process from microscopy image capture to force reconstruction can be quantitatively evaluated (Fig. 2). If the finite-element mesh is small enough compared to pixel size, the simulation allows us to reproduce the substrate deformation caused by cellular traction force in order to generate synthetic images that closely mimic the movement of beads as would be obtained in TFM. Within this framework, we can parametrically explore the effects of any processing step separately and the resulting accuracy of the traction force reconstruction for a known input.

By using these synthetic images as a ground truth, we propose an alternative approach to track cell-induced deformations making use of the Lucas-Kanade optical flow algorithm^13^ with an extension known as Kanade-Lucas-Tomasi (KLT) method to detect locally varying and large deformations using a pyramidal approach^14^. This approach solves the optical flow equation on detected features based on the assumption that the optical-flow is constant within a defined neighborhood. The performance of this approach is compared to the tracking algorithms commonly used in TFM which are based on image correlation calculated either on a regular grid without *a priori* knowledge of particle locations^7^,^15^,^16^ or directly on the previously identified beads^6^,^12^.

Along with this alternative tracking method, we investigated and quantified the influence of critical experimental parameters on the outcome of the TFM analysis. Specifically, we estimated the traction error produced by any choice of the experimental setup (marker bead location and density) with respect to any desired displacement field algorithm.

Using extensive simulations on a wide range of input parameters, we quantitatively assessed the effects of these parameters against a known calibration benchmark. Therefore, the here presented data is a quantitative estimation of how optimal bead location and density offers a high potential to improve both the accuracy and the signal quality of the results and the associated errors that should be expected.

## Results

### Optical flow feature tracking is orders of magnitude faster than PIV and substantially improves traction reconstruction, especially for small adhesions

We modeled traction as uniform horizontal shear stress at a focal adhesion modeled as a circular area with a diameter ranging from 0.5–5 μm, acting on a cubic substrate large enough to be considered as an elastic half-plane. The resulting displacement data were used to generate synthetic TFM images with known bead locations and displacements that were used to validate various TFM methods and approaches.

We compared the Kanade-Lucas-Tomasi (KLT) optical flow tracker to three different correlation-based tracking algorithms that are most commonly used in TFM: The widely employed particle image velocimetry method (PIV^17^,^18^), a template-matching PIV as proposed by Tseng (TPIV^15^) and a correlation-based particle tracker that uses the previously detected particles for the interrogation location (PTV^6^) (Fig. 3a, other densities see supplementary Fig. S1). For all PIV approaches and for the PTV algorithm we used a window size of 16 pixels (=0.96 μm) and for the KLT a window size of 8 pixels (=0.48 μm) and the grid size was always 8 pixels. We measured the relative TFM error as “deviation of traction magnitude” (DTM^6^), where a value of 0 defines perfectly accurate traction reconstruction and −1 complete underestimation of the true traction input.

It was remarkably evident that improved traction reconstruction emerged from the optical flow (KLT) algorithms compared to the correlation-based trackers (PIV/PTV) especially for small focal adhesions, for which the traction error was reduced approximately 40–50%. For large adhesions, the difference was less substantial but still pronounced. Moreover, the signal-to-noise ratio (SNR, defined as the ratio between traction at the adhesion and background) was markedly higher for optical flow compared to current standards for TFM that rely on correlation-based tracking approaches (Fig. 3b).

Graphical representation of the required computational time as a function of evaluation points (features or beads for feature-based methods (PTV & KLT), grid points for PIV) revealed that the methods based on cross-correlation are significantly slower, with the needed time linearly increasing with the number of evaluation features. In turn, the optical flow tracker required minimal time spans independently of the number of features in the images (Fig. 3c). This is in accordance with the fact that the KLT algorithm solves the least-squares problem globally, rather than sequentially for each evaluation feature.

### 2D TFM always underestimates true traction forces, particularly for small adhesions

Since most algorithms for calculating substrate displacements from bead images are based on small interrogation windows around the points of interest (uniform grid position or bead location), each calculated displacement only represents an average of the displacements within that interrogation window. This causes an unavoidable underestimation of traction forces, an effect that increases with increasing window size, as has been previously described^5^,^6^. Therefore, for window sizes >1 pixel, fully accurate reconstruction can only be achieved if the local variations within the interrogation window are negligible.

We demonstrated the relative TFM error (DTM) based on the “best-case” scenario, i.e., a noise-free “true” displacement field derived numerically from the FE solution (Fig. 4a). In order to simulate the displacement resolution and mesh caused by different window sizes, we first calculated the discretized (ideal) displacement field caused by traction on every pixel. Depending on the interrogation window size, the final displacement vector was averaged from displacements within the given interrogation window sampled from the full field. Because the accuracy of the traction magnitude depended on whether or not an interrogation window (and the corresponding interrogation point on the mesh) lies on an adhesion area, we averaged the traction force value of n^2^ interrogation positions that are shifted within the window area, e.g. for a 32 pixel window, the shown value is the mean DTM of 32 × 32 = 1024 unique TFM calculations. A window size of 1 pixel corresponds to the full displacement field, which is practically not achievable using the tracking algorithms presented in this work.

These values represent the upper limit of force reconstruction accuracy (i.e. lower error limit) that can be achieved using 2D TFM on a 3D traction field^6^. Inherently, smaller window sizes and larger adhesion areas decrease the potential error limit. To put it into a different perspective, Fig. 4b shows the same error as a function of adhesion diameter, but is expressed in units of the applied window size (mesh size is 50% of window size, i.e. neighboring interrogation windows overlap by 50%). This fitted curve to the data shows a clear trend toward reduced error for larger FA and smaller window sizes, where we can consider a stable force reconstruction with a DTM (error) below 20% if the window size is approximately 3–5 times larger than the adhesion diameter. This result is similar to that described by Sabass and colleagues where the outcome of the force reconstruction was evaluated based on displacement field analytically derived from continuum material laws^6^. However, in our study we defined the ratio as the size of the FA relative to the applied window size, as opposed to the mesh size. The error difference between a smaller mesh size (window overlap 50%) and a larger mesh size (window overlap 0%) was only apparent for very small adhesions and for large window sizes (green line in Fig. 4b). For windows that were approximately as large as the adhesion size (ratio ~ 1), the error was up to 20% lower using a finer mesh. This is in line with the Nyquist sampling theorem since a finer mesh results in at least two sampling grid points within a focal adhesion. When the window is at least half the size of the focal adhesion or smaller (ratio > 2), using a finer mesh size (i.e. higher window overlap and >4 sampling points) does not further influence the outcome. Therefore using an ideal (continuous, and noise free) displacement field, without tracking any beads, the window size should be at least ¼ of the focal adhesion size of interest to calculate a reasonably accurate force reconstruction (DTM < 20%). For smaller adhesions, the influence of the displacement mesh size becomes more evident and even though it is still possible to measure those forces, the limitations, and high error should be kept in mind during evaluation of the traction patterns.

### Higher density bead distribution on the surface improves spatial resolution

It is generally accepted that the experimental setup used for TFM analysis has a high influence on the achievable result, both in accuracy and quality. In order to further validate the performance of the KLT optical flow tracker, we simulated a wide range of experimental parameters, which are commonly used in TFM.

One of this critical parameters is the choice of fluorescent beads, which need to be accurately tracked to measure the cell-induced deformations. Firstly, we investigated the influence on the traction error when the beads are confined to the surface of the substrate rather than embedded within it (Fig. 5). As expected, the surface bead configuration yielded lower traction errors than the volumetric distribution. However, the difference in the estimated error values calculated for both conditions was surprisingly high, especially in the case of small focal adhesions. In a similar manner, SNR also improved with surface bead distribution, an effect that was most prominent for small adhesions. For lower bead densities, differences between surface and volumetric distributions did follow similar trends but were less pronounced (supplementary Fig. S1). We thus conclude that a sufficiently high bead density – with markers as close as possible to the adhesion – is essential for an accurate traction reconstruction.

Along with their location, the density at which beads are placed within or on top of the substrates has an obvious influence on TFM. Therefore we simulated different bead densities for each approach using the optical flow tracker and surface-distributed beads (Fig. 6; also see supplementary Fig. S2). Increasing the bead density led to an improved force reconstruction. This performance gain was present in both DTM and SNR, however increasing the bead density higher than 5 *beads/μm*^*α*^ (surface distribution: α = 2, volumetric distribution: α = 3) counterintuitively lowered the quality of the reconstructed traction field (as indicated by the SNR). This surprising result could be explained by ambiguous local derivatives within a small window when the bead density is above a certain level. This could lead to spurious displacements in regions with little or no traction, represented by the higher variance in the background and hence the lower SNR value. For volumetric bead distributions and different interrogation window sizes, this trend was not observed. However, since the DTM is still decreased for high bead density and the SNR – though slightly lower – remains high, it is suggested that a bead density as high as possible is one of the design goals of the experimental setup.

To further explore these results, we focused on the bead themselves and their ability to reproduce the real cell-induced deformations. Within the spatial domain of the substrate, the tracked bead displacements provide a basis to generate an analytical approximation of the true substrate displacement field. We investigated approximation accuracy of bead displacement as a function of simulated bead distribution and density, for both beads confined to the surface and for beads volumetrically-embedded within the substrate. This approximation accuracy is represented as the mean ratio of peak displacement in the simulation (at the center of the focal adhesion) to the “maximally displaced bead”, i.e. the displacement of the bead which is closest to the center of the focal adhesion, for 20 different uniquely and randomly generated bead distributions. Hence a higher density of beads is more likely to represent the real displacement field.

The number of beads beneath a focal adhesion footprint increases quadratically with larger FA diameters (Fig. 7a). These simulated results correspond well with the analytical expectation defined as:


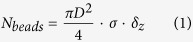


where N_beads_ is the number of beads below a FA, σ the bead density, δ_z_ the thickness of the considered region (focus region, here 1 μm) and D the diameter of the FA. When comparing the surface to the volumetric distributions, there were large differences in approximation accuracy especially for high bead densities (5 & 10 *beads*/*μm*^*α*^), where the accuracy increased from 0.3–0.4 to 0.9–1 (no units) for adhesions up to 3 μm (Figs 7b and c). For lower bead densities (0.33 & 1 *beads*/*μm*^*α*^) the increase was less pronounced but remained almost two-fold. Interestingly for surface distributions, increasing the bead density from 1 to 5 *beads*/*μm*^*α*^ or higher yields a stable and substantially improved performance (Fig. 7c). A similar but less pronounced effect can be observed for volumetric beads but in this case, an increment of the bead density from 5 to 10 *beads*/*μm*^*α*^ can still significantly improve the displacement calculation accuracy (Fig. 7b).

The approximation accuracy is a combination of bead location relative to the center and the displacement field in and around the focal adhesion. The in-plane displacement on the substrate surface that results from a uniform in-plane traction takes the shape of a Bessel function that is invariant relative to the focal adhesion size and displacement peak (Fig. 8a). On the other hand, the depth-variant decrease of the displacement amplitude highly depends on the focal adhesion size and magnitude respectively (Fig. 8b). Therefore in case of a small (<1 μm) focal adhesion, the difference in the relative displacement of a bead located on the surface and a bead occupying a position 0.5 μm below it would be of approximately 50%. If the beads are randomly and uniformly distributed in the volume, the average nearest-neighbor distance of the beads can be approximated by the expected value of the probability function^19^:


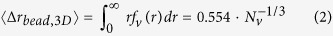


where Δr_bead_ is the expected distance from the center of a bead to its nearest-neighbor and f_v_(r) the probability to find a bead from the center of another bead between r and r + dr. N_v_ is the number of beads per unit volume (bead density). This is also the same distance from any point in the volume (here the focal adhesion center) to the nearest bead location. For the beads confined on the surface, the average nearest-neighbor distance can be approximated as:


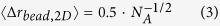


where N_A_ is the number of beads per unit surface (Fig. 8c). The combination of the analytical results of Δr_bead_, which depends on the bead density and distribution (on the surface or within the substrate), and on the displacement field footprint confirmed the simulated approximation accuracy results (Fig. 7c). In order to reach a displacement approximation accuracy of approximately 80%, bead density needs to be high enough to ensure that the bead closest to the center of the FA is located at a distance shorter than 50% of the radius of that same FA, with “surface beads” performing better in that context (Figs 7 and 8).

### Traction magnitude has no influence on error, except for extreme traction levels

Different traction magnitudes from 1% to 50% normalized to the bulk elastic modulus of the substrate were compared for high bead densities (Fig. 9). Increasing the traction from 1% to 10% did not significantly alter the reconstruction error, although the noise ratio was higher for smaller tractions (and thus smaller displacements), particularly when tractions were close to the level of traction background (reconstructed traction noise in areas with no applied traction). Increasing the traction magnitude beyond 10% of the substrate elastic modulus caused substantial problems in displacement tracking as the displacements exceeded the tracking capacity for the algorithm. For lower bead densities, this trend is also visible but less pronounced (see supplementary Fig. S2). This should be considered when designing experiments, as one should tune the experimental substrate to achieve large enough bead movement to clearly distinguish small traction without losing the information at sites of large traction stress.

### Experimental TFM confirms *in silico* results

In order to confirm whether the above-mentioned optimizations also hold for real TFM image sets, we cultured HuO9 osteosarcoma cells on soft polyacrylamide substrates with two different bead distributions and estimated the tractions exerted by the cells using PIV and KLT algorithms (Fig. 10). All trends reported above on the basis of *in silico* TFM data were as well critical in *in vitro* TFM experiments. Independently of the distribution of the fluorescent beads, the estimated displacement field and traction forces (Fig. 10e–l) display higher peak values when calculated with the KLT approach. Comparing the results obtained using surface and volumetric bead distribution reveals higher resolution and less background noise in the surface configuration and a peak traction which is almost three times higher (Fig. 10k and l). In a similar fashion, the peak traction forces analyzed by KLT are approximately twice as high as the PIV derived traction, with a visibly improved background level, especially for the surface beads (Fig. 10j and l).

## Discussion

Measuring cellular traction on flat elastic substrates using TFM is a widely used approach for mechano-profiling of cells and to study the underlying biophysical processes that drive cellular processes such as migration. Nevertheless, the methods used for the estimation of these forces suffer from various limitations. The present work quantifies for the first time how imaging features close to the resolution limit of conventional microscopes, as well as the ill-posed nature of the constitutive laws used to calculate traction forces given displacements, can lead to a very large underestimation of true cellular forces.

Our results clearly demonstrate how potentially catastrophic errors emanate and propagate during sequential steps of a TFM analysis in a manner that may preclude meaningful reconstruction of adhesion traction stresses. We accordingly provide quantitative guidance on “best practices” to minimize such errors. Among our most important recommendations, we suggest that optical flow algorithms (such as the Kanade-Lucas-Tomasi algorithm) be harnessed to minimize tracking errors. We show that these errors are particularly problematic for small focal adhesions and/or in regions of relatively low marker density. These tracking errors are potentially devastating as they occur in the earliest steps of TFM image analysis, with consequences for all downstream analysis. Despite the fact that accurate feature tracking is essential to accurately reconstructing the substrate displacement field, little or no progress has been made in overcoming the performance limits of the particle imaging velocimetry approaches that currently dominate the field. In the present study, we demonstrate that optical flow algorithms offer a high potential to identify small focal adhesions that PIV approaches miss. Also importantly, optical flow algorithms are computationally several orders of magnitude faster (less computationally expensive) than correlation-based PIV approaches. In other words, they are both very fast and more accurate than PIV.

These insights were gained by developing and exploiting a simulation based calibration platform that allows a thorough benchmarking and assessment of all steps involved in TFM. As mentioned above, we focused mainly on the displacement measurement step, a critical link in the chain that has often been neglected. We generated TFM images of fluorescent beads that display high similarity to those obtained experimentally and simulated bead displacements in them by using a finite element solution for applied reference tractions of known shape and magnitude.

This calibration platform improves upon existing simulation environments that have directly focused on force reconstructions downstream of a designated displacement field, an approach that neglects consideration of potentially catastrophic errors that can result from algorithmic bead tracking from experimental data sets^6^,^20^. Firstly, we included the 3D displacement of the beads and discussed the error that accompanies the use of a 2D analysis on images of these 3D substrates, neglecting vertical displacements that are caused even for purely in-plane traction of cells on the substrate surface. Second, including the bead image generation allowed us to test and compare different displacement approaches independently from the force reconstruction. In a similar way, the presented platform can be tuned to simulate any experimental setup by using an exact model for the microscope′s point spread function and the size, density and distribution of the beads.

Starting with several models of experimental traction on a single focal adhesion scale, we quantified the influence of critical experimental parameters such as fiducial bead seeding strategy and focal adhesion size as well as computational parameters during the bead tracking using the standard PIV approach. Using the simulated datasets with known input allowed us to determine the theoretical limits of TFM and to identify potentially avoidable sources of errors. As a result of this analysis, we were able to quantify the potentially critical advantages of using the Kanade-Lucas-Tomasi bead tracker (Fig. 3), as discussed above.

Moreover, the extent to which one can meaningfully analyze lateral traction in a 3D system using only in-plane displacements remains an open point. As mentioned, if the substrate material is incompressible (***v*** ≈ 0.5), the lateral traction/displacement can be decoupled from the vertical displacement. Further, even when ***v*** is substantially lower than 0.5 the error in lateral traction magnitude remains small when neglecting out-of-plane displacements^21^. Similarly, our data show that a very small window size results in an error of almost zero (Fig. 4a), indicating that the vertical displacements indeed have a minor influence. Extending the platform to include vertical displacements (using z-stacks instead of single images) and vertical tractions would allow one to study out of plane traction as well as rotational moments at focal adhesions^20^. While this work may be important for addressing certain specific biological questions^22^,^23^,^24^,^25^, it falls outside the scope of present study.

In order to keep the investigated parameter space and resulting computational datasets manageable, we deliberately implemented certain simplifications to the model. With current imaging systems and the use of beads emitting strong fluorescence, most image noise sources are very small^26^ and hence we neglected its impact. We simulated the influence of photon noise on the bead images and showed that only for a very low photon count the force reconstruction may suffer (see supplementary Note and Fig. S4). Additionally, the traction applied was simulated as being constant over a circular area, whereas it has been observed that the traction peak within a single FA is often strongly fluctuating^3^ while acting on an elliptical footprint, especially for larger sizes. Even though this local variation of traction forces may further complicate accurate reconstruction, the influence of the experimental setup or post-processing is expected to follow a similar behavior, as investigated in this study.

Sub resolution-sized features mimicking the fluorescent beads classically used in TFM experiments were incorporated in the modeled substrates and served as markers of the displacement caused by the tractions. Beads close to the surface, as well with high density, increase the chances to accurately track the displacement field, which was theorized - though not quantified - earlier^6^,^27^. These design parameters become extremely important when looking at small focal adhesions with low traction amplitudes since an undesirable bead distribution (low density, below the surface) does not allow an adequate analysis in the needed resolution level (Fig. 6).

As discussed, using an optical flow tracker can improve traction reconstruction in terms of both error and noise, especially for smaller focal adhesions. In addition, the very low computation time of this approach allows the use of smaller interrogation windows and hence is able to detect features at higher resolutions (Fig. 3)^7^. As suggested by the data presented in this work, using KLT to track beads is likely to outperform correlation based approaches in almost all variants of TFM experiments, including micro-patterned approaches with distinct feature definition^28^. However, it remains to be investigated how KLT performs on images acquired with a widefield microscope and on a substrate with a substantially different refractive index because the larger PSF and increased background signal could disturb the feature detection and hence the tracking accuracy.

Cell-generated tractions are proportional to strain, with limits on accuracy toward both ends of the metric spectrum. In the case of large displacements, assumptions of linear substrate elasticity may no longer hold, while on the other hand, if the cell-induced displacements are too small, tracking accuracy will decrease. Therefore, choosing a substrate material that is optimal for traction measurements that is yet still physiologically relevant is an important task that must be adapted to the target traction and peak displacement of a particular cell type. We simulated different traction force magnitudes as a function of the material stiffness and demonstrated that for stress up until 25% of the material modulus, there was no adverse influence on the result. However, for stresses above this threshold and for large focal adhesions, displacements exceed the ability of the algorithms to track them correctly and thus errors increase. When looking at the peak displacements of the simulated data and the errors caused by tracking the beads, we suggest using a substrate stiffness in order for the peak displacements not to exceed 1–2 μm (supplementary Fig. S4).

Accurately measuring cell-generated traction forces is a challenging task, specifically for microscopy-based, high spatial resolution analysis of the focal adhesions points through which cells act on their surroundings. The presented simulation and calibration framework helps to understand these limits under a given setup and proposes a simple new approach that significantly improves accurate traction force reconstruction, especially for small focal adhesions. We believe that in the future, it will be possible to effectively resolve the complete traction field of a cell in 2D, with our calibration data helping to properly quantify the accuracy of the obtained data and avoid numerical and experimental biases. Hence more reliable conclusions about the interactions between the cell and its immediate surroundings can be drawn.

## Materials and Methods

### Synthetic image generation

**Figure 2:**
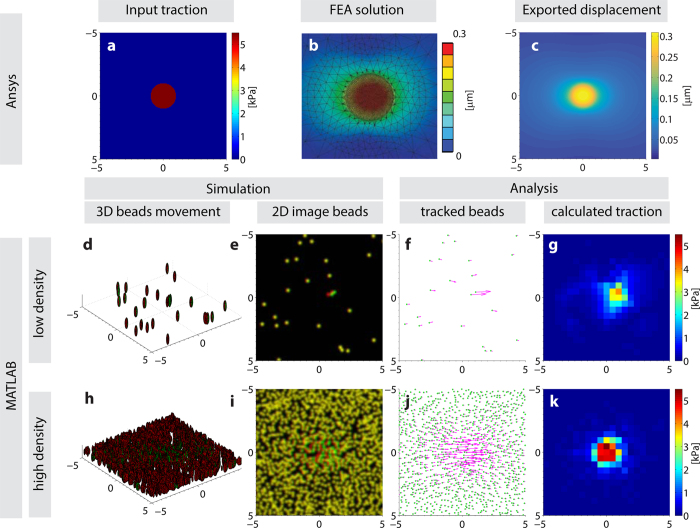
The simulation and evaluation environment operates using three main steps. In the first step (**a**–**c**), a finite element analysis calculates the displacement field of a given traction input (**a**,**b**) which is exported to MATLAB (**c** and **d**–**k**). This deformation is then used to virtually translate 3D beads (**d**,**h**; red: before deformation, green: after deformation), and with user-defined inputs such as bead density (low: **d**–**g**, high: **h**–**k**) and location, simulated traction images can be generated in 2D (**e** and **i**). Using these images, the output of any TFM algorithm can be analyzed (**f**,**g**,**j** and **k**). As a sample result depicted here, a high bead density yields a more accurate force reconstruction.

**Figure 3:**
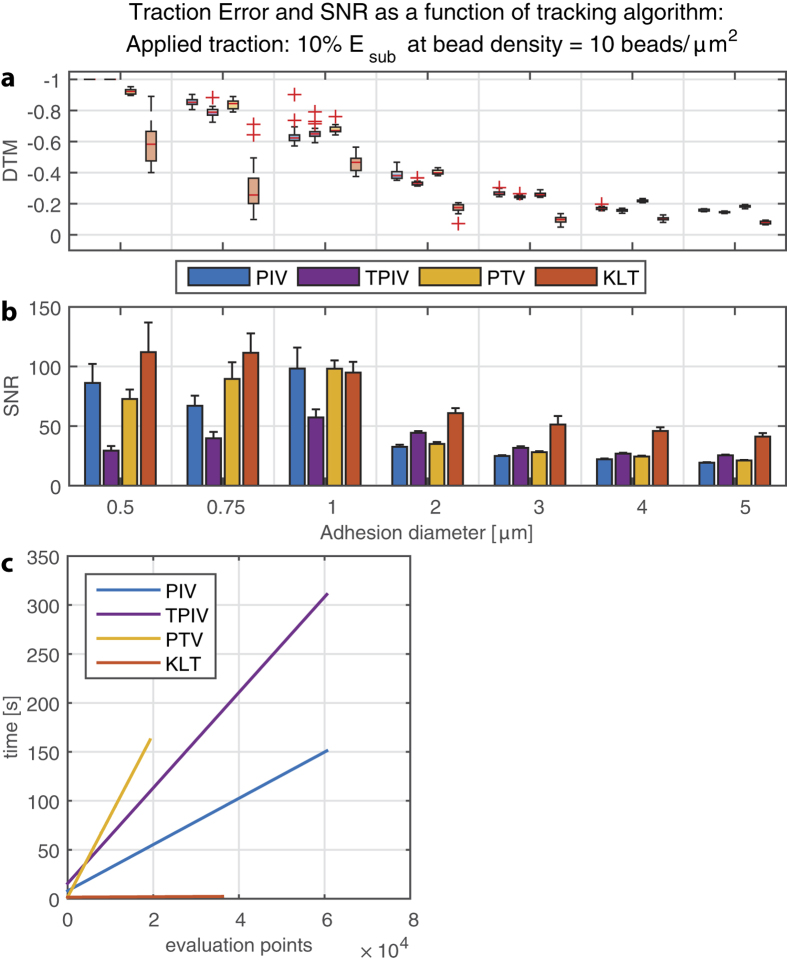
Superior performance of optical flow tracking over standard correlation-based approaches. (**a**) The deviation of traction magnitude (DTM defined as 0 when error free and −1 in the case of complete underestimation (**b**) Signal to noise ratio (SNR) is shown for four different displacement algorithms tested as discussed in methods, shown for the surface (2D) bead configuration. Using KLT to reconstruct the displacements yields a better force reconstruction in both magnitude and quality of the traction images, especially for small adhesions. (**c**) Linear fit to the computation time needed for the displacement analysis as a function of evaluation points within one image. KLT is several orders of magnitude more efficient than correlation-based approaches, also at a large number of evaluation points (beads).

**Figure 4:**
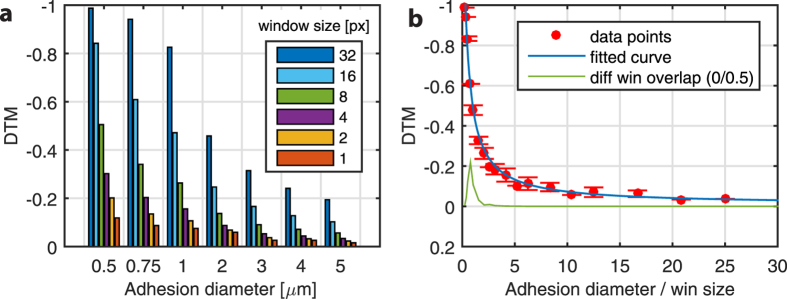
(**a**) Deviation in traction magnitude (DTM) using a simulated displacement field from the FE solution (best-case scenario for force reconstruction) for different interrogationwindow sizes (n^2^). Note that the displacements are averaged within each window to mimic the pre-smoothing by the displacement algorithms. (**b**) The same result, but displayed as a function of adhesion diameter expressed in units of mesh size. A rational function is fitted to the data to show the trend. Adhesions smaller than 4 mesh sizes are difficult to properly reconstruct.

**Figure 5:**
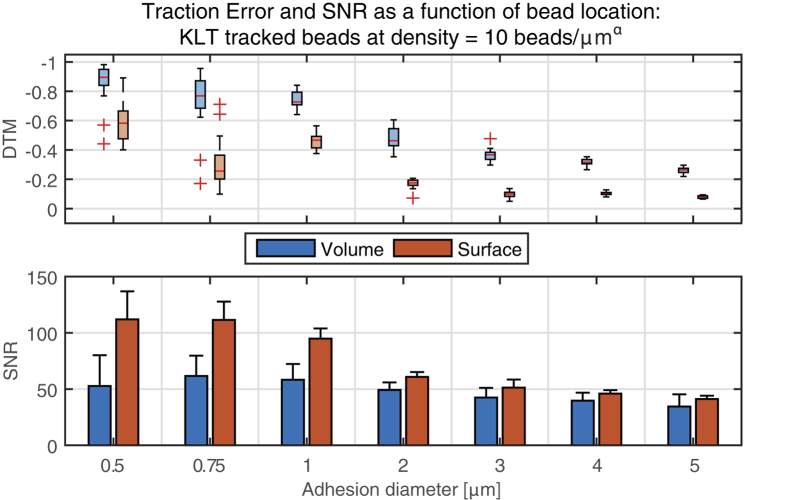
Error, quantified as the deviation of traction magnitude (DTM), and signal to noise ratio (SNR) comparing two different bead distributions: Volumetric beads (3D, α = 3) and surface beads (α = 2). For all focal adhesion (FA) diameters, but especially for smaller ones, using beads on the surface yields a better force reconstruction. All displacements were calculated using KLT with a window size of 8 pixels.

**Figure 6:**
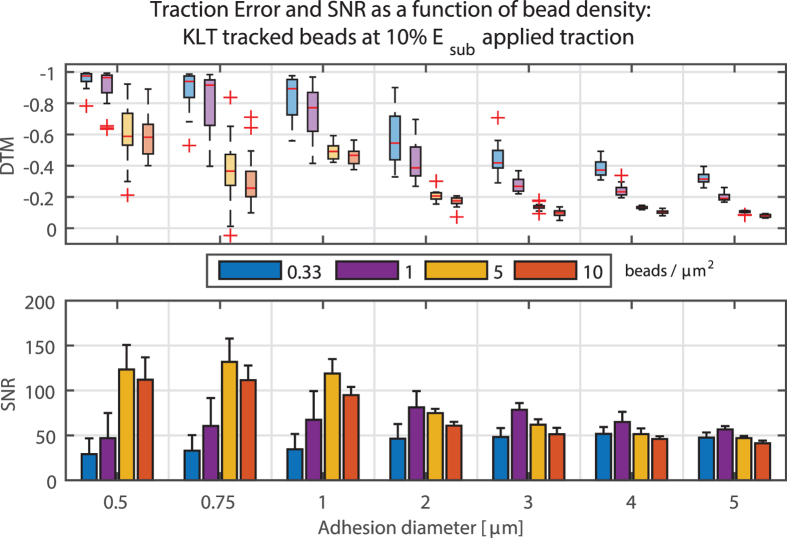
Deviation of traction magnitude (DTM), and signal to noise ratio (SNR) for different bead densities. Up to a bead density of 5 *beads/μm*^2^, the error decreases significantly. A bead density higher than 5 *beads/μm*^2^ only marginally decreases DTM, but also the SNR. All displacements were calculated using KLT with a window size of 8 × 8 pixels.

**Figure 7:**
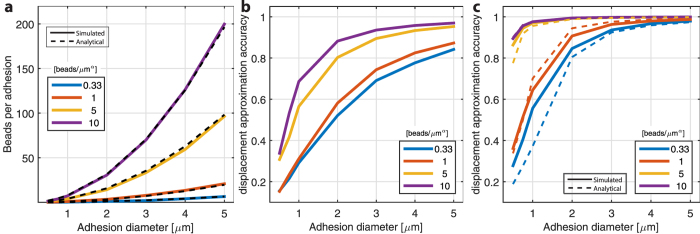
Spatial metrics related to the simulated random distribution of beads in both volumetric and surface bound configurations. (**a**) In both cases, the average number of beads beneath an area of a focal adhesion was evaluated (solid line) shown for volumetric distribution, n = 20; dashed line: Analytical solution per equation (1). (**b**) The bead displacement approximation accuracy of the bead displacement simulation as a function of bead density, shown for the volumetric configuration. This measure is defined as the ratio between the maximum displacements in the FEA simulation to the maximal bead displacement. (**c**) Bead displacement approximation accuracy of the surface bound distribution. Dashed lines represent the analytical solution. For a similar number of beads covered by the focal adhesion, the displacement field is better represented on the surface than the volumetric configuration. In actual TFM measurements, bead marker displacements are used to estimate the true displacement caused by the cell. Therefore, this ratio indicates the ability for a given bead distribution to yield an accurate reconstruction of true displacement.

**Figure 8:**
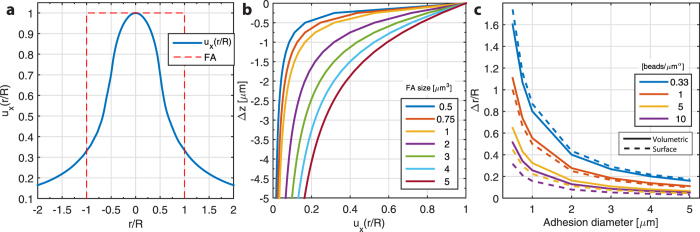
Analytical investigation of the displacements induced by an applied surface traction and the average distance from the adhesion center to the nearest bead. (**a**) Cross-section of an in-plane displacement footprint (u_x_(r/R); blue line) that results from a uniform traction T_x_ on a circular adhesion (red dashed line) as a function of relative distance to the center of the adhesion (r). The displacement can be described by a long-range Bessel function whose shape is invariant to the radius of the focal adhesion (R). (**b**) Normalized displacement magnitude as a function of depth from the surface (z = 0) for different focal adhesion sizes. For small focal adhesions, the displacement magnitude drops rapidly and therefore the bead displacement within the volume of the substrate (z < 0) is only a fraction of the displacement on the surface. (**c**) Analytical result of the average distance from the center of a focal adhesion to the nearest bead location (Δr), normalized to the radius of the adhesion (R). The results are shown for four different bead densities and the two bead distributions (solid line: volumetric distribution; dashed line: Beads confined to the surface of the substrate).

**Figure 9:**
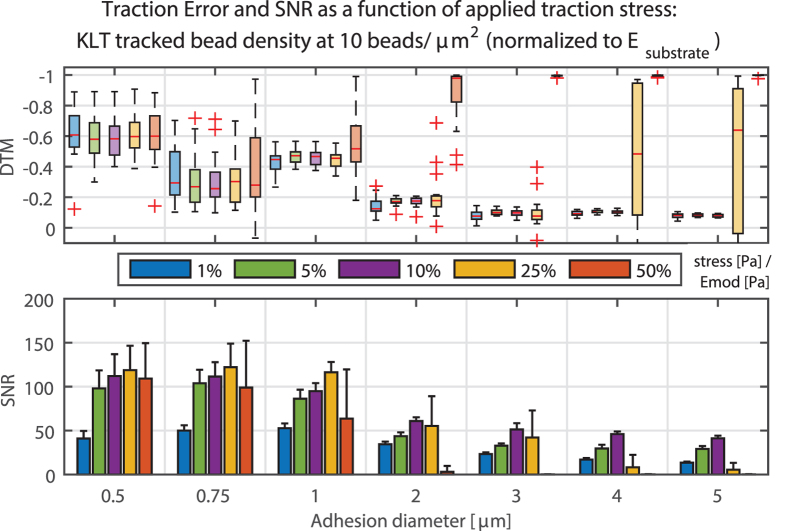
Deviation of traction magnitude (DTM), and signal to noise ratio (SNR) for different traction magnitudes, expressed as a percentage of Young’s modulus of the substrate. Up to a certain magnitude, the influence on the result remains small. However, for large traction and large adhesions, displacements are too big and the tracker fails to correctly match the bead displacements. If these are too small, the signal is of the same magnitude as the background noise, therefore resulting in lower SNR values.

Two main steps are used to obtain engineered microscopy-analog images with simulated tractions. In the first step, we created a finite element model of the substrate to calculate the substrate displacements, which were subsequently used in the image generator for simulating bead displacement in order to generate a set of microscope mimicking images (Fig. 2). We modeled the substrate as a linear elastic cuboid in Ansys (ANSYS Inc., Canonsburg, PA) with dimensions (300 × 300 × 100) μm (x, y, z) where the bottom nodes were held as fixed boundary conditions. We defined the traction as a shear stress, perpendicular to the substrate surface, with a constant magnitude normalized to the elastic modulus of the substrate (5%, 10%, 25% and 50%), acting on a circular area representing the focal adhesion site with diameter ranging from 0.5 to 5 μm^6^,^20^ (Fig. 2a). Since the Poisson ratio is <0.5, there is always a small amount of vertical displacement, resulting in a 3D displacement field. In order to avoid insufficient sampling frequencies, we meshed the body in and around the circular FA area with 50 nm quadratic tetrahedron elements (SOLID187; Fig. 2b). These elements are suitable to account for the local curvature of the focal adhesion and to avoid hourglassing inside the body. After the solution was calculated, we exported the resulting nodal displacements into MATLAB for further simulation and analysis (Fig. 2c).

We generated artificial TFM images with two different bead distributions. In the volumetric configuration, beads were randomly distributed with a given density within the simulated gel. This is usually the case when adding the bead suspension to the stock solution to acquire hydrogels such as Polyacrylamide (PAA) or Poly(ethylene)-Glycol (PEG)^12^,^20^. In the surface configuration, all beads were on the surface of the substrate, as it can be achieved experimentally by either binding the beads covalently using specific cross linkers^29^, or forcing them towards the surface using gravitational pull or centrifugation during polymerization^30^. Surface and volumetric bead densities ranging from 0.33 to 10 *beads*/*μm*^*α*^ (surface distribution: α = 2, volumetric distribution: α = 3) were determined using a sampling volume defined as 60 × 60 × 5 μm^3^ for volumetrically distributed beads and 60 × 60 μm^2^ for surface bead distributions respectively. The lateral dimension roughly corresponds to the field-of-view of a common confocal microscope. The calculated displacement field was interpolated onto the generated bead positions which resulted in sets of reference and deformed bead locations (Fig. 2e and i).

In order to simulate the spherical aberrations that appear due to refractive index mismatch of the immersion liquid and the substrate material used, the thickness of the substrate as well as deviations of the microscope parameters from the design parameters, we used a depth variant scalar point spread function^31^ (see supplemental Note S1). Simulating a spinning-disk confocal microscope, the PSF was approximated as the product between the detection PSF and the convolution between the illumination PSF and the pinhole aperture A^32^:


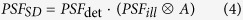


Here we made a valid assumption that the illumination and emission wavelength are similar and therefore *PSF*_*ill*_ ≈ *PSF*_det_^33^. The resulting image *I*(*x, y, z*) was defined as a convolution of the object *I*_*obj*_(*x, y, z*) with the microscope’s PSF as:


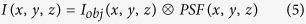


In order to generate mostly general results for using a specific type of microscope, we neglected the influence of noise sources (e.g. Poisson, thermal etc.) that usually appear in imaging processes. To obtain the final bead images for TFM analysis, the top voxel layer of the discrete volume (corresponds to the top focal plane) was saved as a conventional image in Tiff format. All relevant system parameters are summarized in Table 1.

### Displacement Analysis

We propose the use of the Kanade-Lucas-Tomasi (KLT) tracking algorithm, which is a differential approach to estimate the optical flow by the least squares criterion^13^. This approach is based upon the assumption of locally constant flow within the neighborhood of a suitable feature considered^34^,^35^. As for PIV, the problem with large movement is solved using a pyramidal approach^14^. We tested windows in a range of 8–64 pixels, which was possible due to the computational efficiency of the KLT algorithm. Smaller than eight pixels effectively is not applicable, since each bead itself has a (diffraction limited) diameter of approximately 4–6 pixels. In this study, we used the *pointTracker* implementation of the iterative KLT tracker within MATLAB’s Computer Vision toolbox.

### Force Reconstruction

When the cell-induced deformations are sufficiently small, synthetic substrates such as polyacrylamide (PAA) or polydimethylsiloxane (PDMS), which are commonly used cell culture substrates in TFM, can often be assumed linear elastic, homogenous and isotropic^29^,^36^. In such a case, the relationship between the traction forces applied on the boundaries of the substrate **T** and the displacements **U** can be described by an integral solution using a Green’s function:


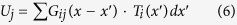


where U_j_ is the displacement (*j* ∈ (*x, y, z*)), T_i_ is the traction force (*i* ∈ (*x, y, z*)) and *G*_*ij*_ is the Green’s function. In this study we only considered in-plane, thus 2D traction forces. Since we modeled the substrate to be thick enough compared to the dimensions of the generated traction, it can be approximated as an elastic infinite half-space and therefore the 2D Boussinesq Greens function can be used^37^. Note that the “true” three-dimensional Greens function is actually a 3 × 3 matrix that also includes vertical contributions to traction and displacements, though in many cases it is safe to assume that the cells are rather flat on the substrate and the generated motion are purely lateral. Most TFM substrates can be characterized incompressible (*v* ≈ 0.5) and thus the lateral and vertical directions can be decoupled. The Green’s function can be solved efficiently in the Fourier domain in an approached called Fourier Transform Traction Cytometry (FTTC^38^) and the solution is found as:


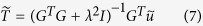


where lambda is the free regularization parameter that has to be determined by data-internal criterion such as the L-curve^39^. In this study, in order to better compare the results caused by different displacement approaches, we kept the regularization parameter lambda at zero. Instead, the noise is minimized for each approach separately using a Gaussian filter for the PIV approaches and a wiener Filter for both PIV and PTV approaches. In order to calculate the Fourier transform efficiently using FFT, the displacement field has to be on a regular rectangular grid. Therefore, all displacements obtained using PTV and KLT were interpolated on a regular grid using the MATLAB *scatteredinterpolant* class with natural neighbor interpolation (an overview of the algorithms and parameters used can be found in supplementary Table S5).

### Traction force microscopy on polyacrylamide gels

Substrates used in our TFM experiments were prepared following a modified version of a previously published protocol^40^. Briefly, a solution containing 4% acrylamide (Sigma A4058), 0.15% bis-acrylamide (Sigma M1533) and carboxylate-modified 200 nm red fluorescent microspheres (5/100; Invitrogen F8810) was prepared and degassed for 15 minutes. Polymerization was initiated by the addition of 2 μl Tetramethylethylenediamine (TEMED, BioRad 7570016) and 10 μl of a 10% ammonium persulfate (APS) solution. Immediately a 6 μl drop was placed on a clean glass surface, covered with a glutaraldehyde functionalized 25 mm diameter coverslip (Menzel Gläser) and allowed to polymerize for 10 minutes resulting in a soft gel with an estimated stiffness of 2.55 kPa +/−0.17^41^.

Next, substrates were immersed in a solution of 10% (3-Aminopropyl)triethoxysilane (APTES; Sigma- Aldrich 440140) in ethanol for 1 hour, followed by 2.5% glutaraldehyde in PBS for 1 hour, and finally carboxylate-modified 200 nm green fluorescent microspheres (1/100 in PBS, Invitrogen F8811) for 1 hour. 1-ethyl-3-(3-dimethylaminopropyl)carbodiimide (EDC, Thermo Scientific 22980) crosslinking was used for the covalent attachment of rat tail collagen type I (Corning 354236) to the carboxy groups of the beads.

**Figure 10:**
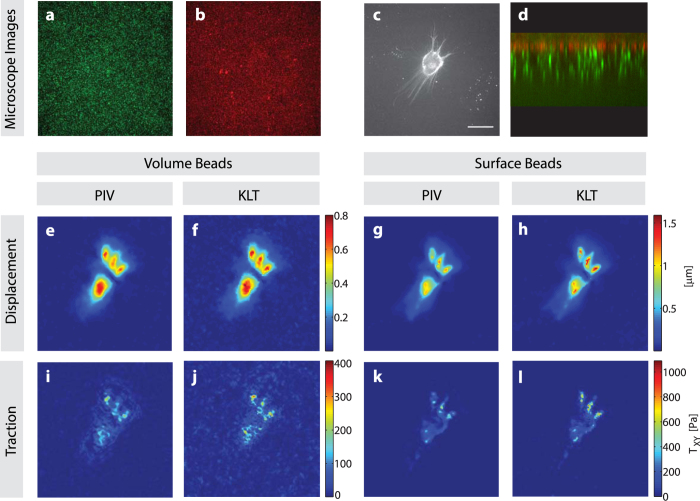
Experimental TFM of a HuO9 cell cultured on top of a polyacrylamide gel with two different bead distributions, and analyzed using two different displacement field algorithms. (**a**) Volumetric bead distribution in the green channel, (**b**) Beads bound on the surface in red channel, (**c**) HuO9 labeled with the lipophilic dye DiD and (**d**) Image composite of the two bead layers in orthogonal view (x-z axis). (**e**–**h**) Absolute displacements in μm, analyzed using PIV (**e**,**g**) and KLT (**f**,**h**) for the volume beads (**e**,f) and surface beads (**g**,**h**). (**i**–**l**) Absolute Traction fields in Pa as obtained using PIV displacements (**i**,**k**) and KLT (**j**,**l**) for volume beads (**i**,**j**) and surface beads (**k**,**l**). Scale bar represents 30 μm.

Substrates were then sterilized by immersion in ethanol, followed by extensive washing steps in PBS and cell culture medium and placed in a 6-well plate. Finally, 6 × 10^3^ HuO9 cells were seeded in each well and let attach to the surface overnight. Before imaging, the coverslips were placed in a metal holder (SKE research equipment) with 0.5 ml complete culture medium and stained with Vybrant DiD (Thermo Fisher V22889) following the recommendations of the manufacturer. For cell detachment 250 μl of a 2.5% solution of SDS in PBS was added to the culture. Image acquisition was performed using a spinning disc confocal microscope (iMic, FEI Munich GmbH) using a 40× N.A. 0.95 objective (Olympus UPLSAPO). Image stacks were analyzed in MATLAB in the same way as the *in vitro* images. First, for both image distribution (Fig. 10a and b), the uppermost stack in focus (corresponding to the attached cell in Fig. 10c) were exported into a single.tif file and subsequently analyzed using the same parameter values as mentioned above for the bead tracking and FTTC, except for the final grid size of the KLT, which was optimized to be at the size 16 pixels.

### Evaluation Metrics

Since the goal was to measure the ability of a routine to calculate traction data from a single focal adhesion of a given size, error metrics were defined that calculate the amplitude of a calculated traction field as well as estimate the noise in the data. Unless otherwise mentioned, the sample size in all simulation was kept constant at 20 samples per condition.

DTM was introduced by Sabass^6^ as a simple relative difference between reconstructed and real traction:


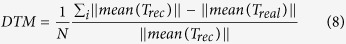


where the sum runs over the averages of all N adhesions and the average of each adhesion is taken on all tractions points within a mask of equal diameter which is located at the adhesion site. A negative value indicates that the calculated traction underestimates the real traction. The Deviation of Displacement Magnitude (DDM) can be calculated the same way.

The ability to detect traction within noisy data is represented by the Signal-to-noise ratio defined as the ratio between traction within the adhesion site and outside as:


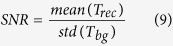


where T_bg_ is the background traction far outside the domain of traction (FA area). The mask for SNR calculation was chosen to be twice the size of the adhesion since the traction footprint mostly appears much wider than the actual footprint.

## Additional Information

**How to cite this article:** Holenstein, C. N. *et al*. High-resolution traction force microscopy on small focal adhesions - improved accuracy through optimal marker distribution and optical flow tracking. *Sci. Rep.* **7**, 41633; doi: 10.1038/srep41633 (2017).

**Publisher's note:** Springer Nature remains neutral with regard to jurisdictional claims in published maps and institutional affiliations.

## Supplementary Material

Supplementary Information

## Figures and Tables

**Figure 1:**
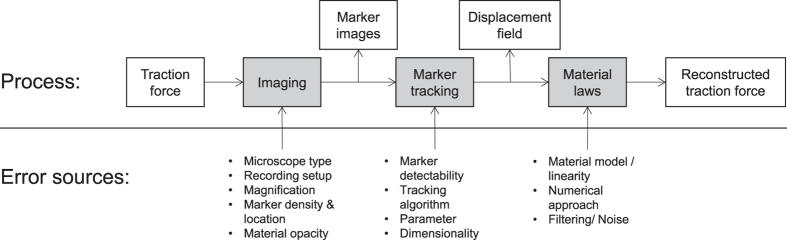
Schematic block diagram of the 3-step TFM process. During each of the three steps (imaging, tracking and force calculation), several experimental and numerical parameters can influence the calculated traction force. Therefore, low-quality imaging and noisy data will inherently lead to an error propagation/-accumulation and deteriorate the quality and accuracy of the resulting traction maps.

**Table 1:** Overview of the simulation parameters used in this work.

Parameter	Value range
**Substrate Thickness**	100 μm
**Substrate mechanical properties**	*E* = 20 *kPa, v* = *0.49*
**Simulated Traction**	5–50% E
**FA diameter size**	[0.5, 0.75, 1, 2, 3, 4, 5] μm
**Bead density**	[0.33, 1, 5, 10] Beads/(μm^3^)
**Bead type**	0.2 μm red-fluorescent beads (emission: 605 nm)
**Bead location**	3D (volumetric), 2D (surface)
**Microscope simulation settings**	Spinning-Disk Confocal with 60 × 1.4 Oil immersion lens. Pixel size (x, y, z) = [0.06 0.06 0.25] μm, ni = 1.51, ns = 1.4, NA = 1.4
**Sample size**	N = 20
