# Supplementary material for: High-resolution traction force microscopy on small focal adhesions - improved accuracy through optimal marker distribution and optical flow tracking
Source: Sci Rep. 2017 Feb 6;7:41633. doi: 10.1038/srep41633 (PMC5292691; doi:10.1038/srep41633)
Supplement: Supplementary Information [file srep41633-s1.pdf]

Scientific Reports

High-resolution traction force microscopy on small focal adhesions - improved accuracy through optimal marker distribution and optical flow tracking

Claude N. Holenstein[1,2,*], Unai Silvan[1,2], Jess G. Snedeker[1,2]

[1]Biomechanics Laboratory, University Hospital Balgrist, University of Zurich, 8008 Zürich, Switzerland,
[2]Institute for Biomechanics, ETH Zurich, 8008 Zürich, Switzerland
* Correspondence should be addressed to J.S. (email: jess.snedeker@hest.ethz.ch)

**Supplementary Figure S1**	Deviation of traction magnitude (DTM) and SNR for different simulation and evaluation criterion
**Supplementary Figure S2**	Deviation of traction magnitude (DTM) and SNR for different simulation and evaluation criterion
**Supplementary Figure S3**	Examples of images and traction analysis done on images with different qualities
**Supplementary Figure S4**	Deviation of displacement magnitude (DDM) for the same dataset and parameter as for the measurement of the deviation of traction measurement (DTM)
**Supplementary Table S6**	Overview of all displacement tracking algorithms and parameters used.
**Supplementary Note**	Point-Spread function Model (PSF)
**Supplementary Note**	Image Noise Model

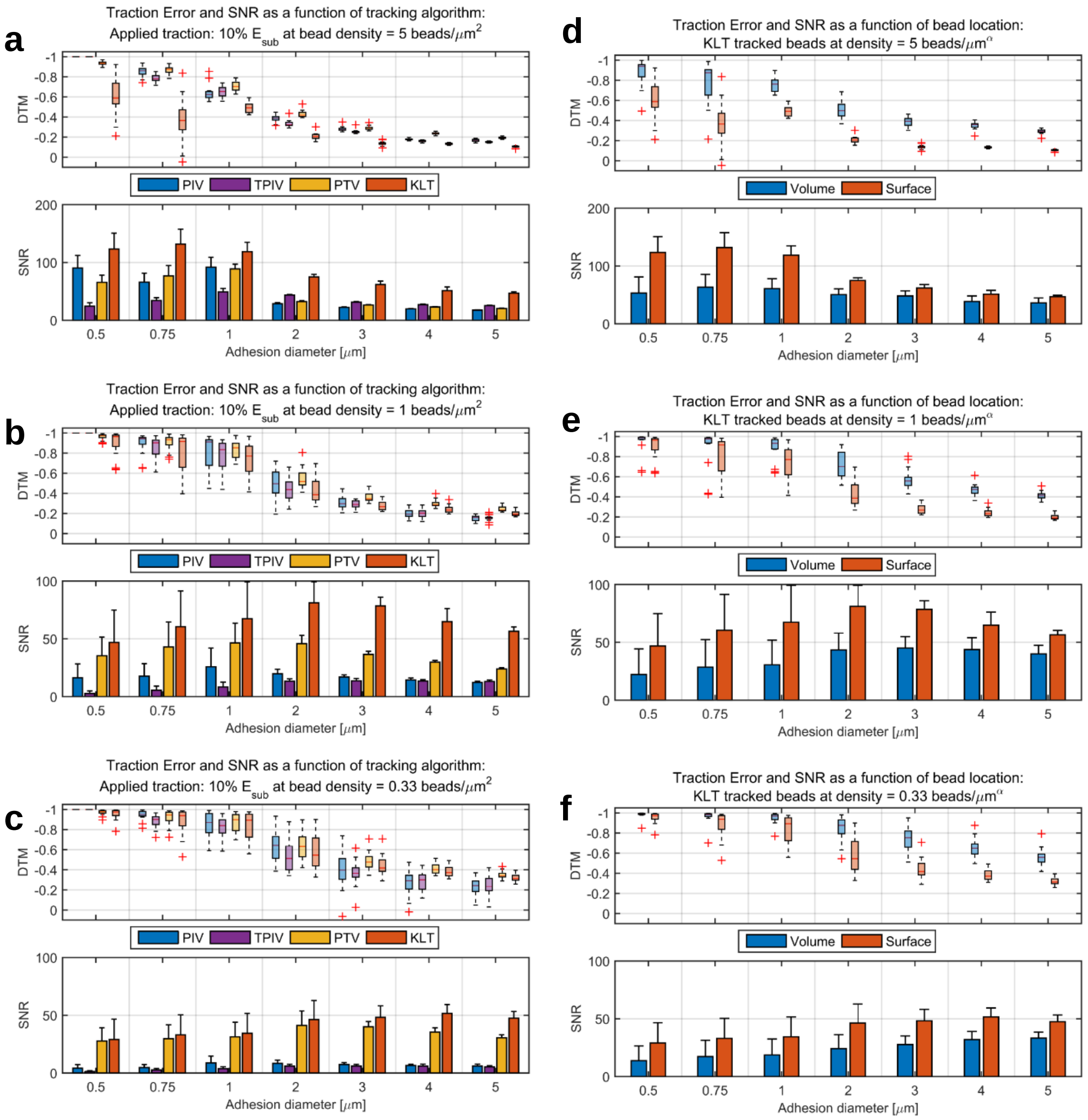

Supplementary Figure **S1**. Deviation of traction magnitude (DTM) and SNR for different simulation and evaluation criterion. (a-c) DTM for different displacement algorithms (as described in Figure 4) for beads distributed on the surface of the substrate with a density of (a) 5, (b) 1 and (c) 0.33 beads/μm^3. (d-f) DTM and SNR comparing surface and volume distribution (as described in Figure 5) for beads distributed on the surface of the substrate with a density of (d) 5, (e) 1 and (f) 0.33 beads/μm^3.

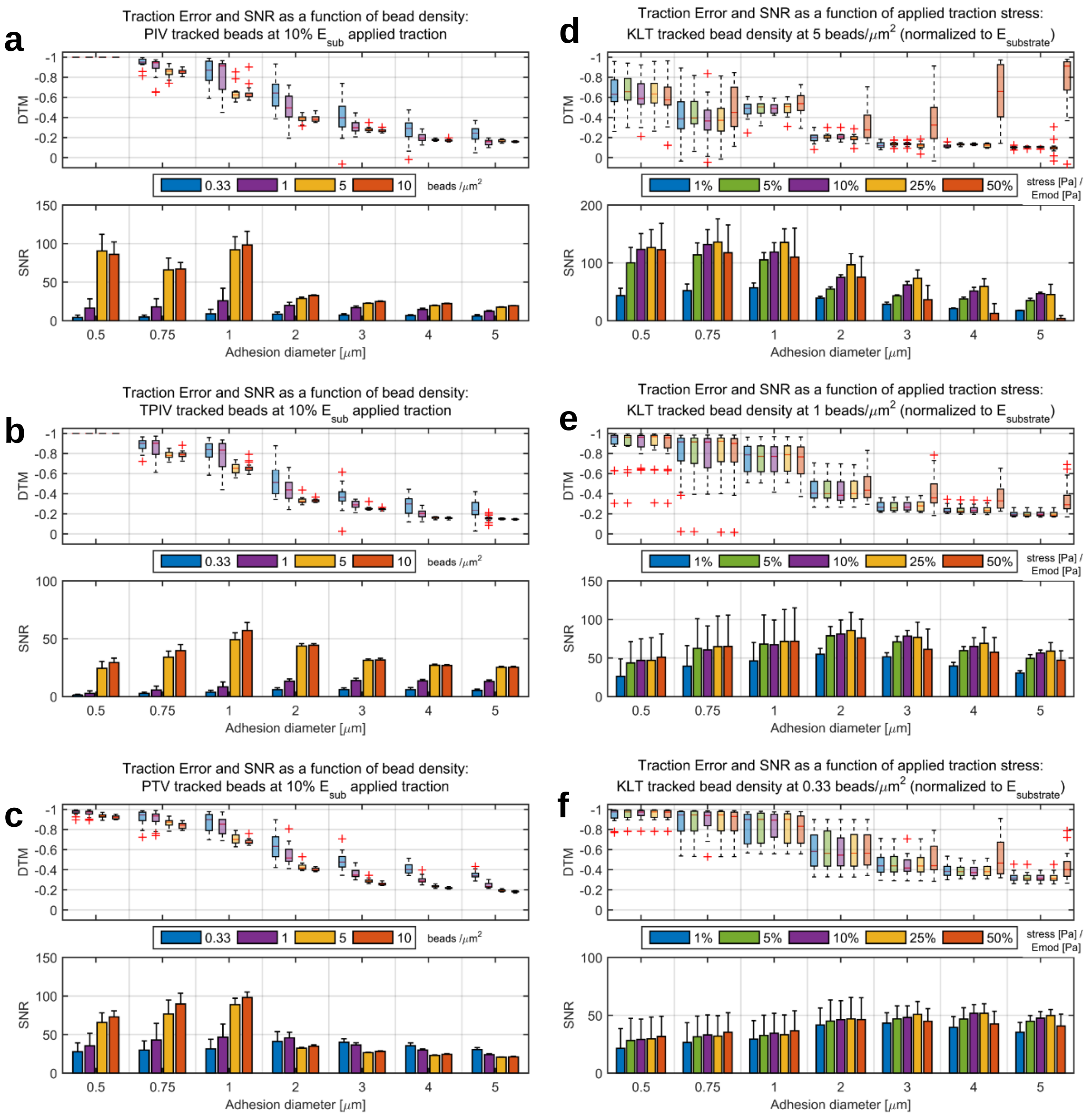

Supplementary Figure **S2**. Deviation of traction magnitude (DTM) and SNR for different simulation and evaluation criterion. (a-c) DTM and SNR for different bead densities (as described in Figure 6), analyzed for (a) conventional PIV, (b) template matching PIV (TPIV) and (c) Particle tracking velocimetry (PTV) as described in material & methods. (d-f) DTM and SNR comparing different input tractions (as described in Figure 7) for beads distributed on the surface of the substrate with a density of (d) 5, (e) 1 and (f) 0.33 beads/μm^3.

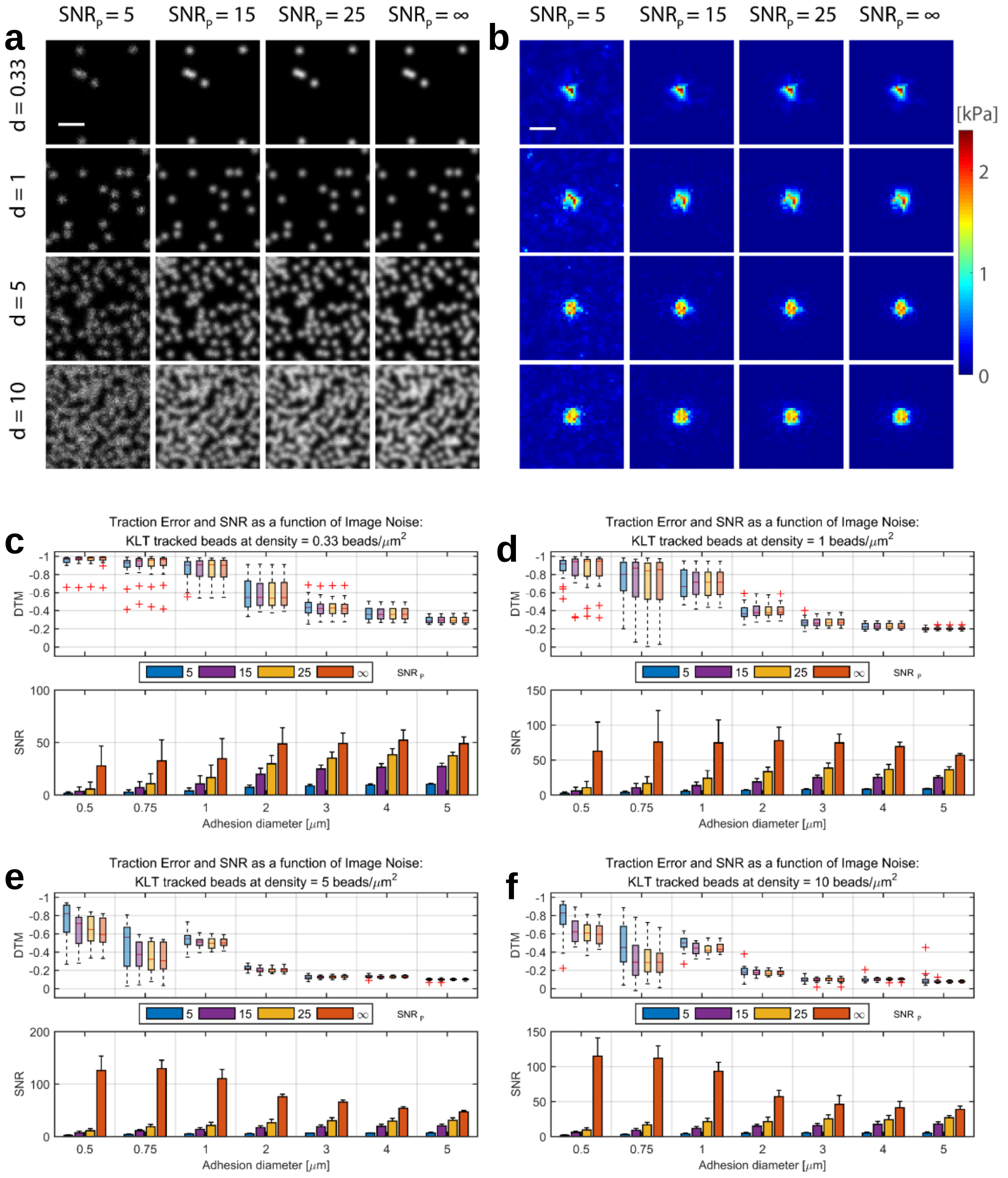

Supplementary Figure **S3**. Examples of images and traction analysis done on images with different qualities. (a) Examples of simulated bead images as used in the simulation with varying densities d (d=0.33 - 10 beads/µm³) and Poisson (shot) noise (SNR_p = 5 – 25, infinity refers to no noise), scale bar 1 µm. (b) Example results for the traction footprint as obtained using KLT and FTTC (as described in materials & methods), scale bar 5 µm, input traction 2kPa. (c-f) DTM and SNR comparing different Poisson noise input SNR_p for (c) 0.33, (d) 1, (e) 5 and (f) 10 beads/µm³.

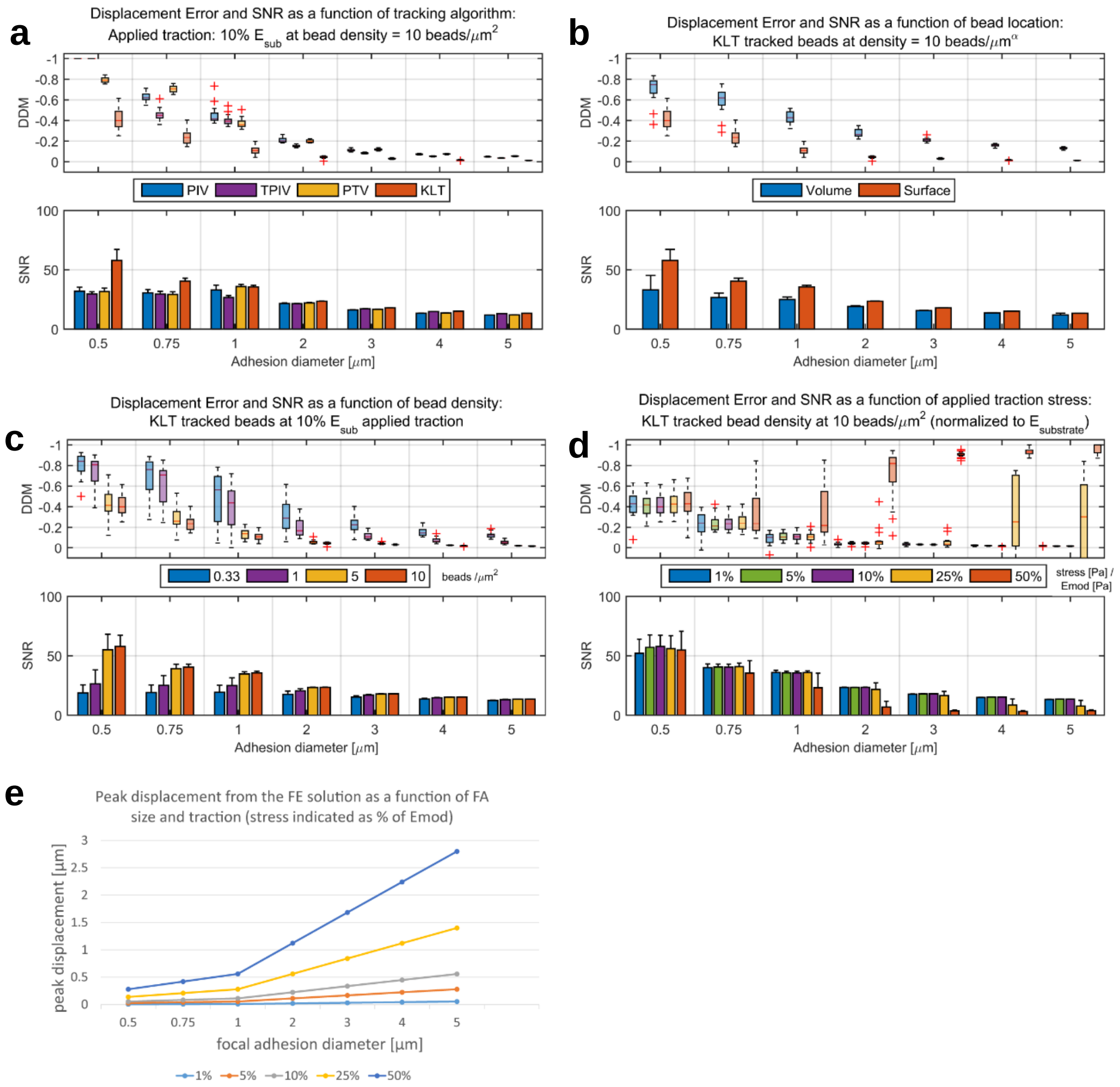

Supplementary Figure **S4**. Deviation of displacement magnitude (DDM) for the same dataset and parameter as for the measurement of the deviation of traction measurement (DTM; Figure (4-7)). (a) DDM and SNR for the four different displacement algorithms tested. (b) DDM and SNR for beads distributed in the volume (3D) and surface (2D) of the measurement substrate. (C) DDM and SNR for different bead densities, distributed on the surface of the substrate (analyzed with KLT). (d) DDM and SNR for different input traction magnitude expressed as a percentage of the young's modulus of the substrate. As the input traction and focal adhesion size increases, the peak displacement increases which cannot be tracked accurately anymore. (e) Peak displacement obtained from the numerical FE solution as a function of adhesion size and focal adhesion diameter.

Supplementary Table **S5**. Overview of all displacement tracking algorithms and parameters used. Bold numbers are final values used to calculate the results in this study.

**Analysis Method**	**Window size (nxn pixel)**	**Grid**	**Vector filter**	**Smooth**	**Traction**
PIV	**16**,32,64	50% of Winsize (**8**,16,32)	After each iteration	Wiener, Gaussian	FTTC
PTV	**16**,32,64	**8**	On final grid	Wiener, Gaussian	FTTC
KLT	**8**,16,32,64	**8**	On final grid	Wiener	FTTC

Supplementary Note: Point-Spread function Model (PSF)

Since we are measuring bead locations through a thick layer of substrate, we used the depth-variant scalar model of Gibson and Lanni (Frisken Gibson and Lanni, 1992) which describes the PSF of a wide-field microscope by the Kirchhoff diffraction integral:

$$PSF(r,z|z_P) = \left| A \int_0^1 e^{iW(\rho,z|z_P)} J_0(krNA\rho)\rho d\rho \right|^2$$

Where k is the wave number of the light, NA the numerical aperture of the objective, Jo is the zero-order Bessel function, $r = \sqrt{(x - x_P)^2 + (y - y_P)^2}$ is the lateral distance to the particle, ϱ the normalized aperture at the objective back focal plane and A is a constant amplitude.

Further is $W(\rho, z) = k \cdot OPD$ where the optical path difference (OPD) accounts for refractive index mismatch of a light ray emitted by a point source located at depth z_p passing through the specimen medium, the coverslip and the immersion medium before reaching the objective. A simplified version of the OPD can be written as:

$$OPD = z_P\sqrt{n_s^2 - NA^2\rho^2} - t_c^*\sqrt{n_{c^*}^2 - NA^2\rho^2} + \left(z_P - z_n + n_c\left(-\frac{z_p}{n_s} + \frac{t_c^*}{n_c^*}\right)\right)\sqrt{n_c^2 - NA^2\rho^2}$$

Where z_n denotes the position of the focal plane, and n_c and n_s the refractive indices of the combined layers (cover-slip and immersion medium combined) and the specimen. The variable t denotes the thickness of the combined layer (working distance of the microscope). The parameters n_c^* and t_c^* are the design values for the refractive index and the thickness of the combined layers. More information can be found in (Aguet *et al.*, 2005, 2008).

The resulting PSF is lateral symmetric and shift-invariant, but not in axial direction.

Supplementary Note: Image Noise Model

In fluorescent microscopy, there are many possible sources of noise during imaging. The most common sources are photon noise (shot noise), dark noise and read-out noise. To test the influence of noise on our simulations, we used a noise model presented earlier (Aguet *et al.*, 2005), where – under the assumption of high-performance CCD or CMOS cameras- only the shot noise is considered. The photon noise is modeled as a Poisson distribution with a mean proportional to the simulated photon count, as simulated using the PSF model. The photon count effectively measured during experiments depends on many parameters. We defined the photon count as a multiplier c to the PSF, i.e.

$$\bar{q} = c \cdot PSF$$

Where $\bar{q}$ is the predicted number of photons according to the level and the observed number of photons q follows the Poisson distribution:

$$P(q) = \frac{\bar{q}^q}{q!} e^{-\bar{q}}$$

It can be shown that the standard deviation of a Poisson distribution is equal to the square root of the average number of events $\bar{q}$, the Shot noise Poisson distribution is defined as:

$$SNR_P = \frac{\bar{q}}{\sqrt{\bar{q}}}$$

Using relevant SNR_P values between 5 and 25 (infinity in case of noise-free), this resulted in values for the multiplier c defined as 5, 225 and 625. The results can be seen in Figure S4 (A), where example bead images for the four SNR_P values and bead densities of 0.33 – 10 beads/µm3 are depicted. Example traction footprint for a 3 µm focal adhesion for these 16 cases are shown in Figure S4 (B). For a SNR_P below 10, the background noise is much more dominant, although the traction footprint did not change significantly. This results can also be seen in the subfigures (C-F), where the numerical results are shown as DTM and SNR (SNR denotes here the ratio of traction on focal adhesion to the background noise, as described in materials & methods). For bead densities 0.33 and 1 beads/µm3, the input noise SNR_P has no significant influence on the traction error only on the output SNR, which is almost zero for small FA. For high bead densities, a low input SNR_P indeed results in a slightly higher DTM, especially for small FA. These results suggest, that for very high imaging noise, traction reconstruction indeed can become complicated on focal adhesion scale, since we often have no a-priori knowledge of the location of the FA. However, for medium to high quality imaging systems ($SNR_P > 5$) the influence of the shot noise to the error in traction of the focal adhesion footprint is very small. Therefore we performed all other tests as presented in this paper without shot noise.

Supplementary References

Aguet, F., Van De Ville, D., and Unser, M. (2008). An accurate PSF model with few parameters for axially shift-variant deconvolution. In: 2008 5th IEEE International Symposium on Biomedical Imaging: From Nano to Macro, IEEE, 157–160.
